# Clinical overview and outcome of the Stuve-Wiedemann syndrome: a systematic review

**DOI:** 10.1186/s13023-022-02323-8

**Published:** 2022-04-23

**Authors:** Hélène Warnier, Christophe Barrea, Sarah Bethlen, Isabelle Schrouff, Julie Harvengt

**Affiliations:** 1 Department of Paediatrics, CHU of Liège, Liège, Belgium; 2 Department of Paediatrics, Neuropeadiatrics, CHU of Liège, Liège, Belgium; 3 Department of Physical Medicine, CHU of Liège, Liège, Belgium; 4 Department of Orthopedic Surgery, CHU of Liège, Liège, Belgium; 5 Department of Human Genetics, CHU of Liège, Avenue de l’Hôpital 1, Sart-Tilman, 4000 Liège, Belgium

**Keywords:** Stuve-Wiedemann syndrome, Skeletal dysplasia, Dysautonomia, Multidisciplinary care

## Abstract

**Background:**

Stuve-Wiedemann syndrome (SWS) is a rare and severe genetic disease characterized by skeletal anomalies and dysautonomic disturbances requiring appropriate care. Peer support is mandatory to fill the lack of clinical recommendations in such rare diseases. We report a new case and provide the first systematic review of all previous published cases.

**Objective:**

To better describe the timeline of SWS and to improve paediatric management.

**Data sources:**

SWS English publications available on Pubmed until 31/03/2021.

**Study selection:**

Case description combining typical osteo-articular and dysautonomic involvement (with 2 items by categories required for children < 2 years and 3 items > 2 years).

**Data extraction:**

Demographic, clinical, genetics and outcome data.

**Results:**

In our cohort of 69 patients, the median age at report was 32 months. Only 46% presented antenatal signs. Mortality rate is higher during the first 2 years (42% < 2 years; 10% > 2 years) mainly due to respiratory failure, pulmonary arterial hypertension appearing to be a poor prognosis factor (mortality rate 63%). After 2 years, orthopaedic symptoms significantly increase including joint mobility restriction (81%), spinal deformations (77%) and fractures (61%).

**Conclusions:**

Natural history of SWS is marked by a high mortality rate before 2 years due to dysautonomic disturbances. A specialized multidisciplinary approach is needed to address these early mortality risks and then adapt to the specific, mainly orthopaedic, needs of patients after 2 years of age. Further research is required to provide clinical guidelines and improve pre-natal counselling.

## Background

Stuve-Wiedemann Syndrome (SWS) (#OMIM 601559) is a rare genetic condition with autosomic recessive inheritance characterized by the association of an osteo-articular involvement and a dysautonomic pattern.

Initially described as lethal in infancy, several cases and series of long-term SWS survivors were then reported [1, 2]. In a such rare disease, improving the clinical syndromic description is essential to optimize the management of these patients with prolonged survival.

The SWS was first described by Stuve and Wiedemann in 1971 [3] but the disease was recognised as an unique condition since 2000: overlap with a lethal form of Schwartz-Jampel Syndrome (SJS) called “SJS Type 2” had been noticed by several authors during the nineties [4–6]. The establishment of the molecular and genetic basis of the disease in 2004 by Dagoneau et al*.* confirmed the unique entity for these two conditions linked to several mutations in the *LIFR* gene (#OMIM 601559), mapped on the 5p13 chromosome [7].

The SWS is characterized by a combination of symptoms with bone involvement and dysautonomic disturbances, therefore the disease is classified in both groups of skeletal dysplasias (subgroup of bent-bone dysplasias) and ciliary neurotrophic factor (CNTF) pathway related disorders [8].

In the last few years some patients with SWS phenotype without *LIFR* mutations were described [1, 9] and, conversely, patients with *LIFR* mutations but incomplete SWS phenotype (eg dysautonomic features but lack of long bones involvement) [10], suggesting a genetic and phenotypic heterogeneity among the syndrome.

We report here the original case of a girl whose daily evolution could be observed in our hospital until the age of 19 months because of parental abandonment. We also conduct the first systematic review of clinical aspects of all previous reported cases of SWS in order to better delineate the clinical presentation and natural history of SWS and to provide guidance to improve management of SWS patients.

## Case report

### Neonatal presentation and diagnosis

The female patient is the first child of consanguineous Gypsy parents. Pregnancy was unfollowed. The only prenatal ultrasonography done 10 days before birth showed short bilateral femora. The girl was born at 41 1/7 weeks with a birthweight of 3430 g (P50), birth length of 48 cm (P10), and head circumference of 35 cm (P75). Apgar score was 9/10. Physical examination showed mild dysmorphic facial features, short and bowed lower limbs, camptodactyly, global hypotonia (without consciousness trouble) and weak sucking reflex.

The neonatal period was marked by transient and mild respiratory distress, recurrent fever with all bacteriological work-up negative. Due to severe swallowing trouble and feeding difficulties (vomiting, low oral intake) an enteral feeding by nasogastric tube was started from birth.

**Fig. 1:**
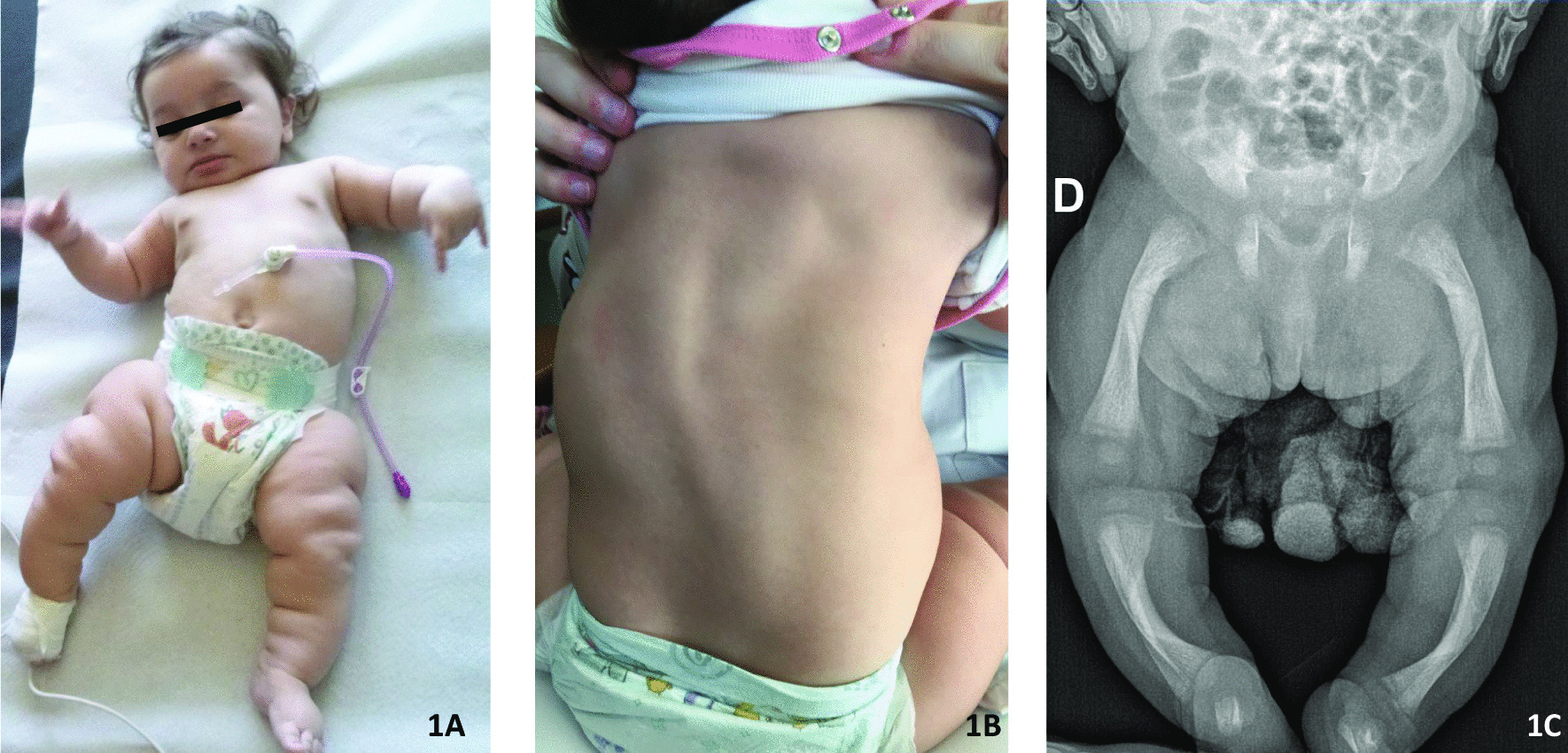
Our case report pictures. **A** Our patient at 8 months of age: no significative dysmorphism, bilateral short and bowed legs. **B** A clinical thoracolumbar scoliosis was observed since the age of 6 weeks, the picture shows the evolution at the age of 14 months. **C** Inferior limb radiology: bowed femur and tibia, diaphyseal cortical thickening at the concavity and wide metaphysis

SWS was rapidly suspected as she met the clinical diagnosis criteria (association of skeletal involvement with short and bowed long bones, and dysautonomic pattern). Whole skeleton radiological study showed typical aspect of long bones (bowed femur and tibia, diaphyseal cortical thickening at the concavity, wide metaphysis with decreased density and abnormal trabecular pattern) (Fig. 1). Diagnosis was confirmed by genetic analysis with homozygous c.756 dup p.(Lys 253*) *LIFR* mutation.

### Osteo-articular evolution

Limbs deformities have been present since birth with bowed legs and joint mobility restriction. Thoracolumbar scoliosis has been observed since the age of 6 weeks. Radiological survey at age of 18 months showed progressive thoraco-lombar scoliosis (42° T4–T12). She received close follow-up by physiotherapist and orthopedist surgeon and benefited from conservative management with daily physiotherapy throughout the paediatric stay, and she has worn a brace since the age of 18 months.

### Non-orthopedic aspects

Hyperthermic episodes were frequent in the first months of life, often associated with common viral infections or during hot summer days. Sinusal tachycardia and excessive sweating were frequently noticed during these episodes. Hyperthermia remained present until discharge at 19 months of age, but episodes after the age of one year are less frequent and less severe.

She had recurrent episodes of severe respiratory distress consecutive to viral infections and she presented two suspected aspiration pneumonias requiring high flow oxygen supplementation.

At age of 13 months she had a specially severe presentation in the course of Respiratory Syncytial Virus infection requiring an invasive ventilation. She developed at this time enterocolitis followed in the next weeks by persistent severe stenosis of ascendant colon and left colic flexure, requiring laparotomy for partial ileocolic resection at age of 17 months.

Persistent feeding and swallowing difficulties since birth led to placement of a gastrostomy tube at age of 2 months. Oral nutrition could be started at 18 months of age with high precaution regarding the risk of aspiration. She also had a smooth tongue and repetitive self-biting lesions after teeth eruption requiring some teeth extractions.

Ocular aspects were marked by hypolacrimation and absent corneal reflex leading to left corneal opacity. She received close ocular follow-up and treatment with artificial tears, ointments and contact lens since the neonatal period.

Neurologic initial evaluation and follow-up showed generalized hypotonia and hyporeflexia with delayed development milestones and reduced pain perception. Cognitive development appears to be quite well related to the patient's living conditions.

### Outcome

She walked at 2 years and she began school at 2 years and 6 months. Shortly after that, unfortunately, she died in the context of an acute respiratory infection having required paediatric intensive management.

## Methods

### Search methods

We conducted a systematic analysis of the medical literature to identify all published clinical cases of SWS using the online database PubMed, until May 31st, 2021. The search query was limited with the terms “Stuve-Wiedemann Syndrome”. All the publications identified were included and analysed. These were supplemented with the incorporation of the secondary references found in each article.

We reviewed each article adhering to the Preferred Reporting Items for Systematic Reviews and Meta-Analysis (PRISMA) individual patient data (IPD) guidelines (Fig. 1) [11].

### Eligibility criteria

We collected clinical case reports written in English.

The clinical cases had to match the definition of the SWS syndrome, combining typical osteo-articular and dysautonomic involvement. The clinical description had to include several data in the two main categories of the symptoms of SWS mentioned above (“osteo-articular” and “dysautonomic”): 2 items had to be reported in each category for case reports of children under 2 years of age, and 3 items in each category for children over 2 years of age.

### Exclusion criteria

Original articles such as review article or other publications that did not contain individual data were not included. The clinical cases that did not match the SWS definition or did not contain enough detailed data in each category (cf supra) were excluded as well as case reports of foetuses. Proved or highly suspected duplicates were also removed.

### Data extraction and analysis

Data was extracted from all the case reports included [1, 3, 4, 7, 9, 12–38]

The demographic information included age, gender, geographic origin, potential consanguinity and outcome (with age at death and cause of death if mentioned). Clinical presentation was analysed under different aspects: antenatal and neonatal presentation, dysmorphology, osteo-articular and non-orthopedic manifestations. The latter category was divided into sub-groups: temperature control, ocular, stomatological, cardio-respiratory and neurological manifestations. Orthopaedic and non-orthopaedic clinical aspects were analysed in two subgroups of patients according to the age at report (< 2 years or ≥ 2 years, named as “long term survivors”).

Each clinical feature is reported as present, absent or not reported/not applicable. Unless we mention a comment, by default the prevalence of each feature (percentage in parenthesis) was defined as the number of patients presenting this feature reported on the total cohort.

## Results

### Demographics data and outcome

**Fig. 2:**
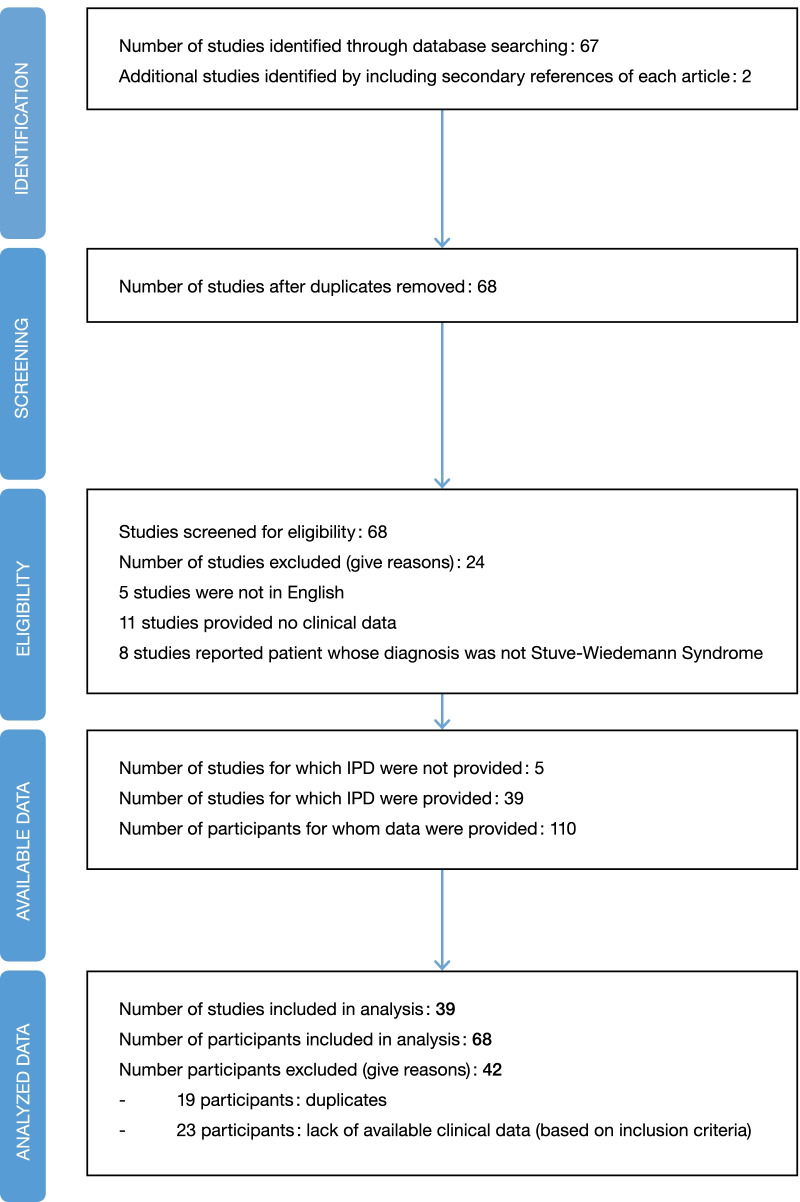
Preferred reporting items for systematic reviews and meta-analysis individual patient data (IPD) flow diagram (based on Stewart et al. [11])

Total cohort includes 69 patients (Fig. 2) with 2 subgroups according to age at report: 38 patients were reported < 2 years and 31 patients ≥ 2 years. Demographic features of the participants are summarized in Table 1. Age of patients at the report varies from a few hours to 27 years. Consanguinity was reported in 40 patients (65% of patients with available data). This high prevalence of consanguinity is consistent with the autosomic recessive mode of inheritance of the SWS. Most of the reported cases came from Europe (51%).

**Table 1:** Details of the demographic features for the cohort (n = 69)

*General features*		
Sex ratio (n = 61)		M/F = 0.97 (NR: 8)
Reported consanguinity (n = 62)		40/62 (65%) (NR: 7)
Affected relatives (n = 69)		38/69 (55%)
Median age at report (25th–75th percentile)		32 months (11.5–72 months)
Geographic origin (n = 69)		Europe 35/69 (51%) Middle East 22/69 (32%) Africa 8/69 (12%) South or central America 4/69 (6%)
*Outcome*		
Mortality rate (n = 69)	Total cohort: 32/69 (46%)	< 2 years: 29/69 (42%) ≥ 2 years: 3/31 (10%)
Age at death (n = 32)	Median (25th–75th percentile): 7.5 months (3–18 months)	*Repartition of death by age category*: Neonatal period (< 1 month): 14/32 (44%) Infancy (1 month–2 years):15/32 (47%) Childhood (> 2 years): 3/32 (9%)
Cause of death (n = 32)	Respiratory failure 14/32 (44%) Hyperthermic episode 9/32 (28%) Sudden death 2/32 (6%) Necrotizing enterocolitis 2/32 (6%) Post-operative 2/32 (6%) Not reported 3/32 (9%)	*Cause of death by age category*: *Neonatal period*: Respiratory failure or PAH: 10/14 Hyperthermic episode or sudden death: 1/14 Other cause: 3/14 *Infancy*: Respiratory failure or PAH: 2/15 Hyperthermic episode or sudden death: 10/15 Other cause: 3/15 *Childhood*: Respiratory failure or PAH: 2/3 Hyperthermic episode or sudden death: 0/15 Other cause: 1/3

Regarding the outcome of the participants, the global mortality rate is 46% in the total cohort with in particular a mortality rate of 42% (29/69) during the first two years of life and of 10% (3/31) after the age of two. Causes of death change substantially according to age: in neonates, most of deaths (10/14 = 71%) are caused by respiratory failure. Pulmonary arterial hypertension (PAH) is associated with a poor prognosis (fatal issue in 5/8 patients = 63%). During infancy (1 month–2 years), most of deaths occur during hyperthermic episodes (10/15 = 67%). Mortality rate decreases drastically after age of two.

### Antenatal and neonatal presentation

In our descriptive cohort, at least one anomaly was detected by antenatal ultrasounds in 46% of patients (Table 2). The most frequent anomaly was short and/or bowed limbs, reported as present before birth in at least 32% of the patients. Intra-uterine growth restriction was found in 17% of patients while a normal prenatal growth is reported in more than half of the cohort. Reduced foetal movements were reported in < 10% of patients. When reported, neonatal adaptation can be either poor or good in quasi equal proportion.

**Table 2:** Antenatal and neonatal features reported in the cohort (n = 69)

	Present	Absent	Not reported
*Presence of any antenatal sign in 32/69 (46%)*			
In utero detection of short/bowed limbs	22 (32%)	32 (46%)	15
Intra-uterine growth restriction	12 (17%)	35 (51%)	22
Oligoamnios	11 (16%)	9 (13%)	49
Reduced fetal movements	6 (9%)	3 (4%)	60
Poor neonatal adaptation	10 (15%)	8 (12%)	51

### Dysmorphology

Table 3 shows heterogeneity of dysmorphic features in SWS*.* Facial dysmorphisms are described in 100% of the patients but with some degree of variability. The most frequent dysmorphic features (defined by prevalence > 20% in the analysed cohort) are listed in Table 3. Other features were less frequently described such as ptosis of eyelid (4 cases), frontal bossing or single palmar creases (9 cases each).

**Table 3:** Details of the dysmorphic features reported in the cohort (n = 69)

Retrognathia or micrognathia	28/69 (41%)
Short wide nose	23/69 (33%)
Low set ears	21/69 (30%)
Lips abnomalities	21/69 (30%)
Pursed mouth	19/69 (28%)
Midface hypoplasia	19/69 (28%)
Square jaw or square face	15/69 (22%)

### Osteo-articular involvement

Table 4 shows the main osteo-articular manifestations of SWS. Short and bowed limbs are reported in all patients, consistently with the definition criterion of SWS. Radiological aspects are typical with bowed long bones (especially tibia and femora) with cortical thickening at the concavity of the bend, enlarged metaphyses with decreased density and an abnormal trabecular pattern. Other osteo-articular manifestations reported in more than the half of the total cohort are camptodactyly (80%), any degree of joint mobility restriction (65%), growth retardation (57%) and feet malposition (51%).

**Table 4:** Osteo-articular characteristics reported in the total cohort and in the two sub-groups (< and ≥ 2 years)

	Total cohort (n = 69)			≥ 2 years (n = 31)			< 2 years (n = 38)		
	Yes	No	NR	Yes	No	NR	Yes	No	NR/NA
Short and bowed limbs	69 (100%)	–	–	31 (100%)	–	–	38 (100%)	–	–
Camptodactyly	55 (80%)	2	12	25 (81%)	2	4	30 (79%)	–	8
Joint mobility restriction	45 (65%)	5	19	25 (81%)	2	4	20 (53%)	3	15
Fractures	21 (30%)	18	30	19 (61%)	12	–	2 (5%)	6	30
Osteoporosis/osteopenia	19 (28%)	2	48	15 (48%)	–	16	4 (11%)	2	32
Spinal deformation	30 (44%)	21	18	24 (77%)	5	2	6 (16%)	16	16
Feet malposition	35 (51%)	9	25	14 (45%)	8	9	21 (55%)	1	16
Growth retardation	39 (57%)	5	25	27 (87%)	1	3	12 (32%)	4	22

Comparison of data in the subgroups illustrates the progression of orthopedic manifestations during childhood. Early manifestations include camptodactyly (79%; n = 30/38 < 2 years) and feet malposition (55%; n = 21/38). The other orthopedic manifestations are worsening with age. An aggravation of joint mobility restriction is noted (53% < 2 years /81% > 2 years), associated with a progression of the long bones deformities. Osteoporosis prevalence also increases in children (11% < 2 years /48% > 2 years) with consecutive fractures reported (5% < 2 years/ 61% > 2 years). Early spinal deformation, as described in our patient during infancy, is relatively rare but appears very frequently in childhood (16% < 2 years /77% > 2 years). Growth retardation can be noticed early in life and is nearly always reported during childhood (32% < 2 years /87% > 2 years).

### Non-orthopedic manifestations

Table 5 describes non-orthopedic features of SWS. All results are presented with comparison of the 2 age-related subgroups (< 2 years/ > 2 years). All patients in the cohort present several dysautonomic manifestations with a wide degree of variability, and variation according to age. In contrast to orthopedic manifestations, dysautonomic features seem to become less prominent with age.

**Table 5:** Non-orthopedic manifestations described in the total cohort and in the two subgroups (< and ≥ 2 years)

	Total cohort (n = 69)			≥ 2 years (n = 31)			< 2 years (n = 38)		
	Yes	No	NR	Yes	No	NR	Yes	No	NR/NA
*Temperature control*									
Hyperthermia	58 (84%)	5	6	31 (100%)	–	–	27 (71%)	5	6
Hypothermia	7 (11%)	6	56	6 (19%)	6	19	1 (3%)	–	37
Poor temperature regulation	32 (46%)	–	37	28 (90%)	–	3	4 (11%)	–	34
Excessive/paradoxical sweating	33 (48%)	4	32	26 (83%)	2	3	7 (18%)	2	29
*Cardio-respiratory manifestations*									
Respiratory distress	56 (81%)	4	9	24 (77%)	4	3	32 (84%)	–	6
PAH	8 (12%)	–	61	1 (3%)	–	30	7 (18%)	–	31
*Ocular manifestations*									
Hypolacrimation	13 (19%)	6	50	8 (26%)	2	21	5 (13%)	4	29
Corneal opacities	18 (26%)	9	42	14 (45%)	4	12	3 (8%)	5	30
Absent corneal reflex	19 (28%)	8	42	15 (48%)	6	10	4 (10%)	2	32
*Stomatological manifestations*									
Smooth tongue	21 (30%)	1	47	19 (61%)	–	12	2 (5%)	1	35
Tongue injury	17 (25%)	3	49	15 (48%)	2	14	2 (5%)	1	35
Poor dentition	22 (32%)	3	44	22 (71%)	3	6	–	–	38
*Neurological manifestations*									
Swallowing trouble	58 (84%)	3	8	30 (97%)	–	1	28 (74%)	3	7
Abnormal perception of pain	30 (44%)	2	37	24 (77%)	2	5	6 (16%)	–	32
Trismus or myotonia	19 (28%)	17	33	11 (36%)	8	12	8 (21%)	9	21
Hypotonia	41(59%)	1	27	14 (45%)	–	17	27 (71%)	1	10
Hyporeflexia	10 (15%)	11	48	8 (26%)	8	15	2 (5%)	3	33
Delayed motor skills	28 (41%)	–	41	26 (84%)	–	5	2 (5%)	–	36
Normal cognitive level	25 (36%)	1	43	24 (77%)	1	6	1 (3%)	–	37
Seizure	11 (16%)	8	50	5 (16%)	3	23	6 (16%)	5	27

Temperature control disturbances are one of the main characteristics of SWS. Hyperthermia is the most frequent manifestation reported early in life with 71% before 2 years and is systematically described in the subgroup of more than 2 years (100%). Hyperthermia was explicitly reported as absent in only 5/69 patients but all had died within the first two weeks of life. Older children rather develop a poor tolerance to natural variation of external temperature (11%/90%), as well as episodes of paradoxical or excessive sweating (18%/83%). Hypothermia was noticed only in one patient < 2 years and 6 patients > 2 years.

Respiratory distress was found in a large majority of patients (81%), with early presentation (84%/77%). Respiratory failure is the leading cause of death during neonatal period (10/14 = 71%). Invasive respiratory support (19/69 = 28%) and prolonged Pediatric Intensive Care Unit (PICU) stays were described in several patients of the cohort, as well as in our patient. Respiratory manifestations seem to decrease in older children (explicitly described as resolved in 19% (N = 6) of > 2 years subgroup), while some cases of persistent bronchial hyperreactivity are described (16% (N = 5) of > 2 years subgroup).

A PAH is described in several patients with early presentation (18%/3%) and a poor prognosis (mortality rate of 5/8 = 63%).

Ocular and stomatological manifestations can occur early in life but become more evident in older infants and children. The main ophthalmologic complications reported are corneal ulcers and consecutive opacities (8%/45%) due to hypolacrimation (13%/26%) and a lack of corneal reflex/corneal anesthesia (10%/48%). Stomatological manifestations included a smooth tongue with lack of fungiform papillae (5%/61%), a poor dentition (71% in > 2 years subgroup) and frequent tongue injury (5%/48%) due to repetitive biting after teeth eruption in children with decreased perception of pain.

Swallowing trouble are quasi constantly described in SWS patients (74%/97%).

Neurological manifestations encountered in SWS include hypotonia (71%/45%), trismus or myotonia (21%/36%), hyporeflexia (5%/26%), abnormal perception of pain with mainly reduced pain sensation (16%/77%), seizure (16%/16%). Neurological evolution in childhood (> 2 years subgroup) is dominated by delayed acquisition of motor skills (84%, n = 26/31) but normal cognitive level (77%, n = 24/31).

## Discussion

**Fig. 3:**
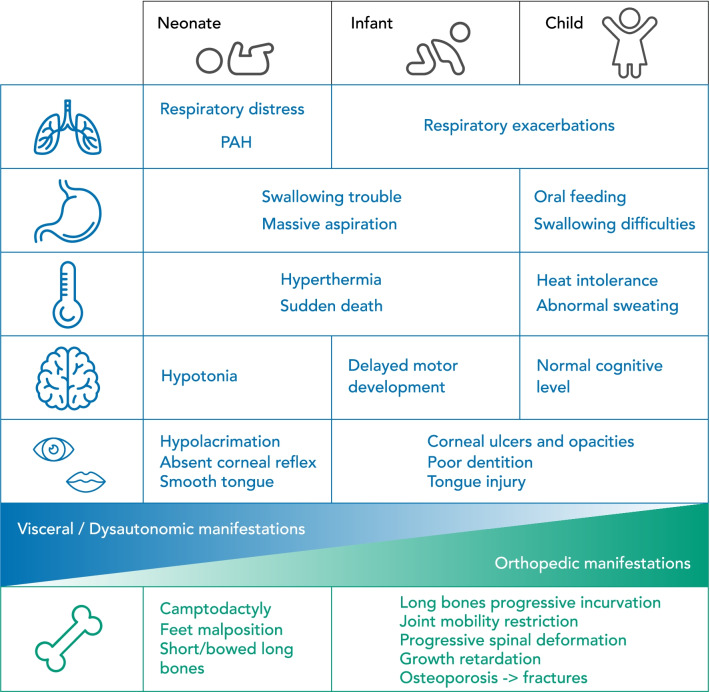
Summary of the disease timeline: clinical evolution for SWS patients before 2 years (Neonate and Infant) and after 2 years of age (represented by the child column). PAH = Pulmonary Arterial Hypertension

Clinical presentation of SWS is defined by the association between orthopaedic aspects and a pattern of dysautonomia. The timeline of the disease is marked by predominant dysautonomic features in infancy with a high mortality rate and prominent orthopaedic aspects in childhood with lower risk of life-threatening episodes (Fig. 3). This review emphasizes the need of a good knowledge of the natural history of SWS to better anticipate age-specific problems encountered by these patients and to offer them the best supportive care management.

### Antenatal

Prenatal abnormalities which are observed in SWS can often only be detected during the late second and the third trimester of pregnancy. In our cohort, at least one prenatal abnormality was found in 46% of the patients. The most frequent prenatal features are mild to moderate micromelia and bowing of the lower limb bones, involving tibia more than femora and generally sparing fibula and upper limbs. Scapulae and pelvis are normal. Another inconstant ultrasound findings include talipes, camptodactyly, intra-uterine growth restriction and oligoamnios [39]. Reduced foetal movements are rarely reported. These few specific and inconstant features explain why antenatal diagnosis of SWS can easily be missed.

Accurate antenatal diagnosis is difficult because of important differential diagnosis with the other bent-bone dysplasias, especially campomelic dysplasia (CD) and kyphomelic dysplasia (KD) which share common skeletal features with SWS [40]. This prenatal distinction between the different osteochondrodysplasias has significant prognostic implications: KD has a good prognosis with regression of skeletal abnormalities and furthermore normal development, since SWS and CD have a poor prognosis with high lethality rate and disabilities in survivors. Involvement of scapulae and pelvis is typical of CD, since enlarged metaphysis and diaphyseal cortical thickening at the concavity of long bones are suggestive of SWS, but a formal diagnosis based on exclusive ultrasound findings remains difficult [41]. A prenatal genetic diagnosis (PGD) can be proposed in such cases (depending on the genetic testing availability). Testing for *LIFR* pathogenic variant should be included in prenatal genetic panels in case of micromelia or bowed long bones, but should also be considered in case of other prenatal abnormalities: intrauterine growth restriction, oligoamnios, or feet malposition, as these anomalies may be the only prenatal finding in some patients with SWS. With the increase of PGD procedures in our medical practice, the diagnosis of SWS will gradually be made more often in the prenatal period. Therefore, our descriptive review is of great significance to provide future parents with more accurate prenatal counselling, especially if the question of TOP (termination of pregnancy) arises.

### Neonatal-infancy

The first two years of life are critical for SWS patients regarding the high mortality rate mainly related to dysautonomic impairment. Osteo-articular manifestations are also present but less disabling. Since prenatal diagnosis can be difficult, diagnosis of SWS is until now often made during neonatal period or early infancy when dysautonomic and orthopaedic manifestations are combined.

Respiratory distress is a major concern in neonates and infants (84%). Respiratory failure is the first cause of death in the total cohort (44% of all deaths), and especially in neonates (71% of deaths during neonatal period). PAH is a poor prognosis factor [23, 30], as illustrated in our cohort with a mortality rate of 63%. Recurrent respiratory distress remains a problem during infancy with frequent exacerbations during common viral infections, often requiring oxygen supplementation, non-invasive or invasive respiratory supports (invasive support reported in 28% (n = 19/69) of our total cohort).

Hyperthermic episodes are another major concern in neonates and infants (71%) and are responsible for most of the deaths after neonatal period (67% of deaths during infancy). Hyperthermia is indeed associated with an increase of sudden death risk [42]. Hyperthermia in SWS is consecutive to dysregulation of temperature control and management is only symptomatic. However, according to good clinical practice, repetitive bacteriological work-up and empiric antimicrobial therapy are frequent in these patients.

The third major concern since birth and during infancy are swallowing trouble (74%) which are probably consecutive to pharyngoesophageal dyskinesia due to an abnormal autonomic control [23]. Nasogastric tube feeding and/or gastrostomy are generally required during infancy to ensure proper feeding and prevent aspiration pneumonia [15, 16, 23]

Other manifestations of autonomic dysfunction are reported since early infancy with ocular and stomatological involvement. SWS patients often show a combination of reduced corneal and blinking reflexes, hypolacrimation and corneal anaesthesia, leading to keratitis, corneal ulcerations and opacities. Close follow-up and early intervention are recommended to prevent definitive lesions and risk of amblyopia. Management includes artificial drops and ointments or surgical procedures (punctual occlusion, lateral tarsorrhaphy, optical iridectomy). Balance between conservative and surgical procedures must be done for each patient [22].

SWS patients have smooth tongue. Reduced pain perception leads to a high risk of tongue biting injury with teeth eruption. Special appliances can be used to cover the teeth to prevent biting lesions.

Regarding psychomotor development, hypotonia and hyporeflexia are common, and motor milestones are generally delayed. The age of walking is among the missing data of our cohort. Progressive limbs deformities contribute to motor difficulties. Early management with supportive physiotherapy is highly recommended and is part of the multidisciplinary approach in addition to the orthopaedic and neurological aspects.

Dysmorphisms in SWS patients are variable and often mild to moderate. Some of these features are consecutive to autonomic dysfunction, such as the pursed mouth (myotonia), corneal opacities, or malposition of finger and toes.

Skeletal findings are also present since birth although dysautonomic manifestations dominate the clinical picture at this age. The main features found in neonates and infants are short and bowed legs (100%) and often camptodactyly (79%) and/or talipes equinovarus (55%). Early vertebral deformities, as present in our patient, are relatively rare (16% < 2 years).

### Childhood

In patients surviving beyond infancy, orthopaedic problems become prominent, as illustrated by the analysis from our “long term survival” group. Children with SWS develop progressive incurvate on of lower limbs and joint mobility restriction [32]. Spinal deformities also worsen with age (77% in the subgroup of > 2 years). Growth retardation is observed in a high majority of children (87% in the subgroup of > 2 years). Osteoporosis and spontaneous fractures are frequently described. Occult fractures are described related to the reduced perception of pain in SWS [1, 27, 32]. Due to this combination of deformities and bone fragility, some of the children become wheelchair dependent during childhood [27, 43]. Early orthopaedic management and intensive multidisciplinary care are needed to try to prevent this adverse outcome. Conservative and preventive management include physiotherapy and the use of braces. Calcium, vitamin D and bisphosphonates [18, 44] can be indicated to prevent osteoporosis and fractures. Even with careful and early orthopaedic follow-up, repetitive surgical procedures are generally needed to correct limbs and spinal deformities. Surgical stabilization of scoliosis is often required, while osteotomies are performed to reduced limbs deformities [45, 46]. Regarding osteotomies, Wright et al. [45] recommend the use of telescopic intramedullary rodding (Fassier-Duval rods) to reduce the risk of recurrence and the need for multiple surgeries.

Long-term SWS survivors often undergo multiple surgical procedures, and there was concern of an increased risk of malignant hyperthermia during general anaesthesia in these patients. However, several authors have reported multiple anaesthesia in SWS without any complication [24]. This is also supported by our new report, our patient having undergone repetitive anaesthetic procedures without complication.

Dysautonomic manifestations remain present in childhood but less severe and mortality rate drops drastically after two years of age. Hyperthermic episodes become less frequent after the first year of life, but temperature instability remains with poor tolerance to natural variation of external temperature and sweating anomalies (paradoxical or excessive sweating) [44]. Swallowing difficulties improve generally during the second or third year of life [20, 23], allowing to introduce progressive oral nutrition and drop out gastrostomy or tube feeding. However high precautions regarding aspiration and choking are needed until at least the age of 5 years and even later. Respiratory exacerbations persist in a minority of children. Ocular and stomatological aspects must be closely followed during childhood [18], as mentioned above.

Finally, from our data, an essential issue is cognitive development which is described as normal in all but one of the reported cases in SWS patients. This concern must be taken into account for these patients in medical ethics discussions where life expectancy and care management are at stake.

Missing data for a part of the analysed item in our cohort are related to the high clinical heterogeneity description of the previous available clinical reports. As a rare disease, SWS must continue to be better described to obtain the more accurate delineation of the clinical picture. To optimize sharing information, standardization of clinical description for further SWS reports would be needed. Based on our data, we suggest a framework for further reports with the main features of the syndrome (Table 6).

**Table 6:** Suggested framework for further reports

*Antenatal*
Short and bowed long bones Oligoamnios Intra-uterine growth restriction
*Dysmorpology (facial)*
*Jaw aspect*: micrognathia, retrognathia, square jaw *Global face shape:* midface hypoplasia, square face *Lips and mouth aspect*: pursed mouth, thin lips *Ears position*: low-set ears *Nose aspect:* short, wide, anteverted nostrils *Eyes:* ptosis of eyelid
*Osteo-articular involvement*
*Long bones:* short and bowed (mandatory criterion for diagnosis) *Hands and feet*: feet malposition, camptodactyly *Articulations:* joint mobility restriction, joint deformation *Bone mineralization:* osteoporosis, fractures *Spinal deformation* (type and severity) *Growth retardation* (weight, height)
*Dysautonomic involvement*
*Temperature control*: hyperthermia, temperature instability, abnormal sweating *Cardio-respiratory:* pulmonary hypertension, respiratory distress *Ophtalmologic*: hypolacrimation, corneal reflex, corneal injuries *Ears/nose/throat*: smooth tongue, tongue injuries, dentition anomalies *Neurological:* swallowing trouble, abnormal perception of pain, myotonia or trismus, hypotonia, hyporeflexia, motor and cognitive development, seizure
*For each reported idem* Explicitly report as present or absent Age of occurrence Age of resolution if applicable (Proposed management)

In the context of rare diseases, coordinated care is currently tending to be more efficient with the implementation of dedicated care structures and medical networks. Multidisciplinarity in the case of SWS requires the coordination of different paediatric specialists as well as surgeons, geneticists, dentists, ophthalmologists, etc.… This kind of follow-up is a real challenge both for the medical aspects and for the support to parenthood: the psychosocial impact on the family can be very severe, leading in our case to parental abandonment.

This review highlights that both neonatal and childhood stage in SWS patients require an intensive and early multidisciplinary care approach. A good knowledge of the disease and its evolution is required to improve management and to provide the best quality of life for each SWS patient. Through this work, we propose for the first time a summary of the disease timeline as a practical tool for the clinicians (Fig. 2).

## Conclusion

SWS is a rare and severe genetic condition due to pathogenic variants in *LIFR* gene. Clinical diagnosis criteria includes the association of skeletal involvement with short and bowed long bones, and a dysautonomic pattern. Systematic review of the reported SWS patients and daily observation of our patient allow us to better delineate the clinical picture of SWS. Symptoms begin early in life with a high mortality rate in neonates and infants due to dysautonomic involvement. PAH appears to be a poor prognosis factor. However, in children surviving beyond infancy, mortality rate drops drastically, dysautonomic pattern becomes less severe and orthopaedic aspects are the principal concern with spinal deformations, osteoporosis and increased risk of fractures. Cognitive development is not altered. Early, specific and coordinate multidisciplinary care are required to prevent and manage complications in these patients and provide them with the best quality of life.

Finally, as a rare disease, SWS requires to be better described through the works of international collaborations to offer care provider dedicated clinical recommendations and better knowledge to improve prenatal counselling.

## Data Availability

All data generated or analysed during this study are included in this published article.

## Abbreviations

SWS: Stuve-Wiedemann syndrome
PAH: Pulmonary arterial hypertension
CNTF: Ciliary neurotrophic factor
PRISMA: Preferred reporting items for systematic reviews and meta-analysis
IPD: Individual patient data
CD: Campomelic dysplasia
KD: Kyphomelic dysplasia
PGD: Prenatal genetic diagnosis
TOP: Termination of pregnancy
